# The Brazil SimSmoke Policy Simulation Model: The Effect of Strong Tobacco Control Policies on Smoking Prevalence and Smoking-Attributable Deaths in a Middle Income Nation

**DOI:** 10.1371/journal.pmed.1001336

**Published:** 2012-11-06

**Authors:** David Levy, Liz Maria de Almeida, Andre Szklo

**Affiliations:** 1 Population Sciences, Department of Oncology, Georgetown University, Washington, District of Columbia, United States of America; 2 Brazilian National Cancer Institute (INCA), Rio de Janeiro, Brazil; University of Southern California, United States of America

## Abstract

**Background:**

Brazil has reduced its smoking rate by about 50% in the last 20 y. During that time period, strong tobacco control policies were implemented. This paper estimates the effect of these stricter policies on smoking prevalence and associated premature mortality, and the effect that additional policies may have.

**Methods and Findings:**

The model was developed using the SimSmoke tobacco control policy model. Using policy, population, and smoking data for Brazil, the model assesses the effect on premature deaths of cigarette taxes, smoke-free air laws, mass media campaigns, marketing restrictions, packaging requirements, cessation treatment programs, and youth access restrictions. We estimate the effect of past policies relative to a counterfactual of policies kept to 1989 levels, and the effect of stricter future policies. Male and female smoking prevalence in Brazil have fallen by about half since 1989, which represents a 46% (lower and upper bounds: 28%–66%) relative reduction compared to the 2010 prevalence under the counterfactual scenario of policies held to 1989 levels. Almost half of that 46% reduction is explained by price increases, 14% by smoke-free air laws, 14% by marketing restrictions, 8% by health warnings, 6% by mass media campaigns, and 10% by cessation treatment programs. As a result of the past policies, a total of almost 420,000 (260,000–715,000) deaths had been averted by 2010, increasing to almost 7 million (4.5 million–10.3 million) deaths projected by 2050. Comparing future implementation of a set of stricter policies to a scenario with 2010 policies held constant, smoking prevalence by 2050 could be reduced by another 39% (29%–54%), and 1.3 million (0.9 million–2.0 million) out of 9 million future premature deaths could be averted.

**Conclusions:**

Brazil provides one of the outstanding public health success stories in reducing deaths due to smoking, and serves as a model for other low and middle income nations. However, a set of stricter policies could further reduce smoking and save many additional lives.

*Please see later in the article for the Editors' Summary*

## Abstract 2

David Levy and colleagues use the SimSmoke model to estimate the effect of Brazil's recent stronger tobacco control policies on smoking prevalence and associated premature mortality, and the effect that additional policies may have.

## Editors' Summary

**Background:**

Tobacco kills up to half its users—more than 5 million smokers die every year from tobacco-related causes. It also kills more than half a million non-smokers annually who have been exposed to second-hand smoke. If current trends continue, annual tobacco-related deaths could increase to more than 8 million by 2030. In response to this global tobacco epidemic, the World Health Organization has developed an international instrument for tobacco control called the Framework Convention on Tobacco Control (FCTC). Since it came into force in February 2005, 176 countries have become parties to the FCTC. As such, they agree to implement comprehensive bans on tobacco advertizing, promotion, and sponsorship; to ban misleading and deceptive terms on tobacco packaging; to protect people from exposure to cigarette smoke in public spaces and indoor workplaces; to implement tax policies aimed at reducing tobacco consumption; and to combat illicit trade in tobacco products.

**Why Was This Study Done?:**

Brazil has played a pioneering role in providing support for tobacco control measures in low and middle income countries. It introduced its first cigarette-specific tax in 1990 and, in 1996, it placed the first warnings on cigarette packages and introduced smoke-free air laws. Many of these measures have subsequently been strengthened. Over the same period, the prevalence of smoking among adults (the proportion of the population that smokes) has halved in Brazil, falling from 34.8% in 1989 to 18.5% in 2008. But did the introduction of tobacco control policies contribute to this decline, and if so, which were the most effective policies? In this study, the researchers use a computational model called the SimSmoke tobacco control policy model to investigate this question and to examine the possible effect of introducing additional control policies consistent with the FCTC, which Brazil has been a party to since 2006.

**What Did the Researchers Do and Find?:**

The researchers developed Brazil SimSmoke by incorporating policy, population, and smoking data for Brazil into the SimSmoke simulation model; Brazil SimSmoke estimates smoking prevalence and smoking-attributable deaths from 1989 forwards. They then compared smoking prevalences and smoking-attributable deaths estimated by Brazil SimSmoke for 2010 with and without the inclusion of the tobacco control policies that were introduced between 1989 and 2010. The model estimated that the smoking prevalence in Brazil in 2010 was reduced by 46% by the introduction of tobacco control measures. Almost half of this reduction was explained by price increases, 14% by smoke-free laws, 14% by marketing restrictions, 8% by health warnings, 6% by anti-smoking media campaigns, and 10% by cessation treatment programs. Moreover, as a result of past policies, the model estimated that almost 420,000 tobacco-related deaths had been averted by 2010 and that almost 7 million deaths will have been averted by 2050. Finally, using the model to compare the effects of a scenario that includes stricter policies (for example, an increase in tobacco tax) with a scenario that includes the 2010 policies only, indicated that stricter control policies would reduce the estimated smoking prevalence by an extra 39% between 2010 and 2050 and avert about 1.3 million additional premature deaths.

**What Do These Findings Mean?:**

These findings indicate that the introduction of tobacco control policies has been a critical factor in the rapid decline in smoking prevalence in Brazil over the past 20 years. They also suggest that the introduction of stricter policies that are fully consistent with the FCTC has the potential to reduce the prevalence of smoking further and save many additional lives. Although the reduction in smoking prevalence in Brazil between 1989 and 2010 predicted by the Brazil SimSmoke model is close to the recorded reduction over that period, these findings need to be interpreted with caution because of the many assumptions incorporated in the model. Moreover, the accuracy of the model's predictions depends on the accuracy of the data fed into it, some of which was obtained from other countries and may not accurately reflect the situation in Brazil. Importantly, however, these findings show that, even for a middle income nation, reducing tobacco use is a “winnable battle” that carries huge dividends in terms of reducing illness and death without requiring unlimited resources.

**Additional Information:**

Please access these websites via the online version of this summary at http://dx.doi.org/10.1371/journal.pmed.1001336.

- The World Health Organization provides information about the dangers of tobacco (in several languages), about the Framework Convention on Tobacco Control, and about tobacco control in Brazil
- The Framework Convention Alliance provides more information about the FCTC
- The Brazilian National Cancer Institute (INCA) provides information on tobacco control policies in Brazil; additional information about tobacco control laws in Brazil is available on the Tobacco Control Laws interactive website, which provides information about tobacco control legislation worldwide
- More information on the SimSmoke model of tobacco control policies is available in document or slideshow form
- SmokeFree, a website provided by the UK National Health Service, offers advice on quitting smoking and includes personal stories from people who have stopped smoking

## Introduction

Since 1989, Brazil has implemented policies to increase cigarette taxes, require bold warnings on cigarette packages, ban many tobacco marketing practices, and generally expand tobacco control programs [1]–[3]. In 1996, Brazil was made a World Health Organization Collaborating Center for the Tobacco or Health Program, thus strengthening its international importance and pioneering role in providing support for preventive actions related to tobacco control to low and middle income nations.

Monteiro et al. [2] documented a steep decline in smoking prevalence in Brazil from 34.8% in 1989 to 22.4% in 2003, but did not attempt to isolate the role of tobacco control policies from long-term trends in smoking prevalence. More recent data [3] indicate that smoking rates have fallen by almost half since 1989, to a level of 18.5% in 2008. The reasons for this steep decline have not been documented.

Most statistical studies have examined the effect of only one or, at most, two tobacco control policies (e.g., [4]), because the ability to distinguish the effects of different policies on smoking rates is limited. Computational models combine information from different sources to explore how the effects of multiple public policies might unfold over time [5],[6].

This paper uses the well-tested SimSmoke tobacco control policy model [7]–[19] to isolate the effect of tobacco policies from previous trends in smoking prevalence. This simulation model projects smoking prevalence and smoking-attributable deaths (SADs) from 1989 forward. Besides examining the role of past policies, the model can be used to consider the potential effect of policies not yet implemented. The Brazil SimSmoke model is used to show the effect of policies implemented between 1989 and 2010, as well as the effect of a set of additional future policies consistent with the World Health Organization's Framework Convention on Tobacco Control (FCTC).

## Methods

SimSmoke includes population, smoking, SAD, and policy modules [6], with the mathematical equations and assumptions provided in Text S1. The model begins in 1989, before major tobacco control policies were implemented. The initial population is divided into smokers, never smokers, and former smokers by age and gender.

To model behavior from 1989 forward, population growth is projected forward through birth and death rates. Data on population, mortality rates, and fertility rates by individual age group and gender are from the Brazilian Institute of Geography and Statistics.

Smoking prevalence is projected forward through smoking initiation, cessation, and relapse rates employing a discrete first-order Markov process (i.e., dependent on current, but not past, rates); individuals are classified as never smokers from birth until they initiate smoking or die, may transition from current to former smoker through cessation, or may return to smoker through relapse. The likelihood of relapse depends on the number of years since quitting. Smoking prevalence for the base year is from the National Survey of Health and Nutrition, a nationally representative household survey of health issues in Brazil conducted in 1989 (*n* = 17,920 households). Because data on former smokers were not available by years since quitting, we use data from a Netherlands 1996 survey [15], in which current and former smoking rates were found to be similar to those in Brazil in 1989. Netherland rates were found to fit better than US rates and validated well, as described below.

Because of empirical challenges in measuring initiation and cessation, and in order to ensure internal consistency of the model, initiation rates at each age are measured in SimSmoke as the difference between the smoking rate of that age and the smoking rate in the last year for the previous age. We allow initiation through age 29 y, when smoking prevalence rates begin to level off. Cessation is tracked after age 29 y. Since cessation data were not available for Brazil in 1989, we use the 1996 Netherlands data on current smokers and those who quit <12 mo ago to estimate cessation rates. The initial cessation rates were about 5% of the smoking population, before relapse. Because relapse data were not available for Brazil, we use US rates [20],[21].

The primary outcome modeled is premature deaths due to smoking, as measured by SADs. Mortality rates by age and gender are first calculated distinguished by smoking status (current, former by years since quitting, and never) by applying relative risks and smoking rates to overall mortality rates. SADs are then calculated by applying the excess mortality risks—measured as the difference between the estimated mortality risk of current (or former) smokers and of never smokers—to the number of current and former smokers (distinguished by years since quitting). Large-scale studies of the relative risk of smoking were not found for Brazil. Because Brazil has a smoking history similar to that of the US, the model uses relative risk estimates for former and current smokers from the US Cancer Prevention Study II [22]–[24], as used in two previous studies for Brazil [25],[26]. Two Brazil studies obtained lung cancer risks comparable to those in the US [27]–[29].

### The Role of Policies

Policy effect sizes are in terms of constant percentage reductions. They are directly applied to smoking prevalence in the year in which the policy is implemented and applied to initiation and cessation rates in future years if the policy is sustained. Unless synergies are specified, the effect of a newly implemented policy is reduced by (1 – the effect of previously implemented policies), thereby bounding the effect of the policies between 0% and 100% and allowing for some offset of simultaneously implemented policies (i.e., the effect of a policy is reduced proportionally by the effect of previously implemented policies). The studies of policy effect size are primarily for high income countries. While the Brazilian economy has progressed substantially since 1989, Brazil was a middle income nation through much of the time period analyzed, and, as such, the policy effects from high income countries are adjusted to reflect differences in health awareness and the degree of urbanization [16]. Effect sizes for each policy were originally based on the advice of an expert panel and thorough reviews of the literature, and modified based on Brazilian policy studies and review by a panel of tobacco control experts in Brazil. In addition, the model has been validated in 19 countries and four US states [7]–[19] exhibiting a wide variety of tobacco control policies; projected adult smoking prevalence rates have generally been within 10% of survey rates. Nevertheless, since the policy studies vary in depth and consistency across policies, we consider uncertainty in effect sizes, based on the variability found in the better studies [11],[16]. To capture uncertainty for smoke-free air laws, marketing restrictions, health warnings, and media campaigns, the bounds are set at 50% above and 50% below the estimated effect sizes [11],[16]. For cessation treatment programs, bounds are 50% below but 100% above the effect size, reflecting the potential to improve treatment through better follow-up of smokers [30]. For youth access restrictions [11], bounds are from no effect to 50% above the effect size, reflecting that many studies find no effect. For price/tax effects, we adopt a lower bound 25% below and an upper bound 25% above the effect size [11],[16], reflecting the greater consistency of price/tax studies.

The effect of each policy depends on its initial level (e.g., the incremental effect of a complete work site smoking ban is less when a nation already has a partial work site ban). Because changes in policy affect the future path of smoking prevalence in SimSmoke, we track policy levels from the year that the model begins, 1989, to 2010. The level of policies is based on information from MPOWER [31], a World Bank report [1], the 2008 Global Adult Tobacco Survey (GATS) [32], and tobacco control staff and organizations whose objective is to collect recent figures on tobacco control in Brazil. Policies and potential effect sizes are summarized in Table 1.

**Table 1:** Policies, description, and effect sizes of Brazil SimSmoke.

Policy	Description	Potential Percentage Effect^a^
**Tax policy**		
Actual prices from 1989–2010, tax changes after 2010	Cigarette price index adjusted for inflation, taxes measured in absolute terms	For each 10% price increase: 6% reduction ages 15–17, 4% reduction ages 18–24, 2% reduction ages 25–34, and 1% reduction ages 35 and above
**Smoke-free air policies (first four policies are additive)**		
Worksite total ban	Ban in all areas	9.0% reduction
Restaurant total ban	Ban in all indoor restaurants in all areas	3.0% reduction
Bar and pubs ban	Ban in all indoor areas of bars and pubs	1.5% reduction
Other places total ban	Ban in three of four: malls, retail stores, public transportation, or elevators	1.0% reduction
Enforcement and publicity	Government agency is designated to enforce and publicize the laws	Effects weakened by as much as 50% if no enforcement and publicity
**Mass media campaigns (policies are mutually exclusive)**		
Highly publicized campaign	Campaign publicized heavily on TV (at least 2 mo of the year) and at least some other media	3.25% reduction (doubled when accompanied by other policies)
Moderately publicized campaign	Campaign publicized sporadically on TV and in at least some other media, and a local program	1.8% reduction (doubled when accompanied by other policies)
Low publicity campaign	Campaign publicized only sporadically in newspaper, billboard, or some other media	0.5% reduction (doubled when accompanied by other policies)
**Marketing bans (first three policies are mutually exclusive)**		
Comprehensive marketing ban	Ban is applied to television, radio, print, billboard, in-store displays, sponsorships, and free samples	10.0% reduction in prevalence, 12.0% reduction in initiation, 6.0% increase in cessation
Total advertising ban	Ban is applied to all media: television, radio, print, and billboard	6.0% reduction in prevalence, 8.0% reduction in initiation, 4.0% increase in cessation
Weak advertising ban	Ban is applied to some of television, radio, print, or billboard	2.0% reduction in prevalence and initiation only
Enforcement and publicity	Government agency is designated to enforce the laws	Effects weakened by as much as 50% if no enforcement
Warning labels (policies are mutually exclusive)		
Strong	Labels are large, bold, and graphic	4.0% reduction in prevalence and in initiation, 10.0% increase in cessation
Weak	Laws cover less than 1/3 of package, not bold or graphic	1.0% reduction in prevalence and initiation, 2.0% increase in cessation
**Cessation treatment programs**		
Complete availability and reimbursement of pharmacological and behavioral treatments, quit lines, and brief interventions	NRT provided in stores without Rx, bupropion provided by Rx, provision of treatments in all health facilities, quit line, 100% smoker brief interventions with follow-up	6.75% reduction in prevalence, 55% increase in cessation
**Youth access restrictions (policies are mutually exclusive)**		
Strongly enforced and publicized	Compliance checks are conducted regularly, penalties are heavy, publicity is strong, vending machine and self-service bans	30.0% reduction for age <16 in prevalence and initiation only, 20.0% reduction for ages 16–17 in prevalence and initiation only
Moderately enforced	Compliance checks are conducted sporadically, penalties are potent, little publicity	15.0% reduction for age <16 in prevalence and initiation only, 10.0% reduction for ages 16–17 in prevalence and initiation only
Low enforcement	Compliance checks are not conducted, penalties are weak, no publicity	3.0% reduction for age <16 in prevalence and initiation only, 2.0% reduction for ages 16–17 in prevalence and initiation only

a Unless otherwise specified, the same percentage effect is applied as a percentage reduction in the prevalence in the initial year and as a percentage reduction in the initiation rate and a percentage increase in the cessation rate in future years. The effect sizes are shown relative to the absence of any policy. They are based on literature reviews, advice of an expert panel, and model validation.

NRT, nicotine replacement therapy; Rx, prescription.

Between 1989 and 2010, Brazil implemented strong taxes, marketing restrictions, health warnings, and other tobacco control programs. Adjusting for general price inflation, cigarette prices doubled by 1998, and were 2.3 times their 1989 level by 2010, largely because of higher taxes. Restrictions on cigarette advertising were first mentioned in 1988, increasing to broad marketing coverage by 2005. Educational programs were implemented in 1996, and have been strengthened to include local programs and strong media campaigns [1]. These programs included policies directed at providing access to cessation treatments. Weak warnings were required on cigarette packages starting in 1996, with the law modified in 2001 to require graphic warnings that covered 100% of the back of the carton. In addition, Brazil strengthened smoke-free air laws in 1996 and 2000, and some cities have implemented a ban on smoking in all enclosed public places since 2007.

### Calibration, Validation, and Model Outcomes

The model estimates smoking prevalence and SADs for the tracking period from 1989 to 2010, and projects future outcomes for 2011 through 2050. The model was validated for smoking prevalence over the period 1989–2008.

The primary data sources for validation and calibration were two national household surveys conducted in Brazil as part of larger surveys: the Brazilian module of the 2003 World Health Survey [1],[3] and the 2008 Brazilian GATS [32] The 2003 survey included a probabilistic sample of 5,000 households. The GATS [3] used a global standardized methodology and a multi-stage stratified sample design, and has 39,425 completed interviews.

We used the tracking period to validate current and former smoking rates by age. Specifically, we compared the predicted current smoking rates from SimSmoke to annual smoking rates by age and gender from the 2003 and 2008 surveys, and the former smoking rates from the model to those from the 2008 GATS [32].

To consider the effect of all policies implemented since 1989, we first set policies through 2010 to their 1989 levels to obtain the counterfactual smoking rates in the absence of the post-1989 policies. The difference between the smoking prevalence with polices at 1989 levels and the smoking prevalence with actual policies implemented yields the net effect of policies implemented since 1989. For the role of single policies, we compared the scenario with only that policy implemented to the counterfactual policy scenario. Because the effects of policies are assumed to be multiplicative, the reduction attributed to each individual policy is relative to the summed effect of all policies. The impact of policies on deaths was estimated by subtracting the number of SADs with policies implemented from the simulated number of SADs with policies kept at 1989 levels.

We also consider the effect of implementing a set of policies consistent with the FCTC, as specified in MPOWER reports [16]. To examine the potential effect of FCTC-consistent future policies, we compare the status quo case, where tobacco control policies are maintained at their 2010 level, with scenarios of stricter tobacco control policies, all implemented in 2011 and maintained in future years.

## Results

### Validation: Predictions of Smoking Prevalence from 1989 to 2008

**Figure 1:**
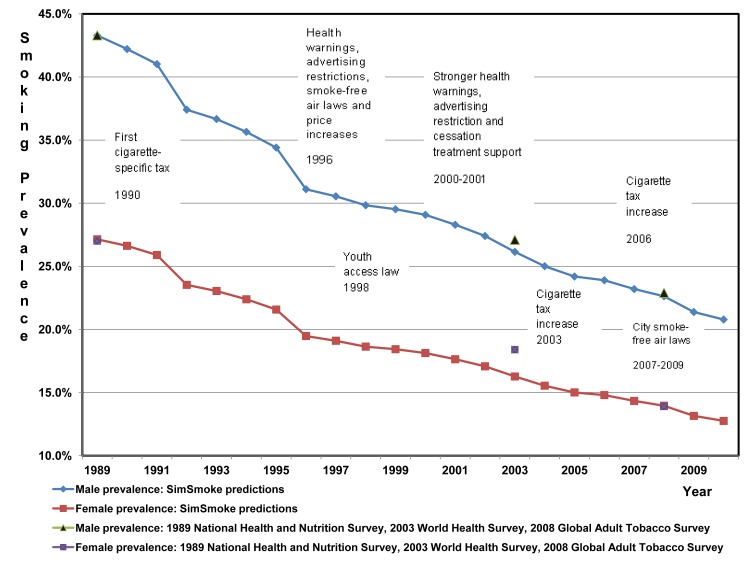
Brazil smoking prevalence for individuals aged 18 y and above, 1989–2010: SimSmoke predictions and various surveys.

Between 1989 and 2008, SimSmoke predicts that the male smoking rate (ages 18 y and above) decreases from 43.3% to 22.9%, approximately a 47% decline in relative terms, and that the female smoking rate falls from 27.0% to 13.9%, approximately a 48% relative decline. As shown in Figure 1 and Table 2, these predictions are very close to the 48% relative decline for males and 49% relative decline for females comparing 2008 to 1989 survey data. As shown in Table 2, SimSmoke is within 2% accuracy for both genders between 1989 and 2008, but is less accurate for the 1989 to 2003 and 2003 and 2008 sub-periods. SimSmoke predicts well by age groups for males by 2008, except for underestimating the decline for the age group 18–24 y. SimSmoke does less well for females, where it underpredicts for the age group 25–44 y and overpredicts for the age group 45–64 y in the 2003–2008 sub-period.

**Table 2:** Validation of the Brazil SimSmoke: predictions versus survey estimates, 1989–2008.

Age	Male Prevalence						Female Prevalence					
	1989	2003	2008	Percentage Change 1989–2003	Percentage Change 2003–2008	Percentage Change 1989–2008	1989	2003	2008	Percentage Change 1989–2003	Percentage Change 2003–2008	Percentage Change 1989–2008
**Smoking prevalence from surveys** a												
18–24	34.1	21.9	14.8	−35.8%	−32.4%	−56.6%	24.2	13.7	9.0	−43.4%	−34.3%	−62.8%
25–44	48.4	27.9	22.6	−42.4%	−19.0%	−53.3%	31.4	21.6	14.4	−31.2%	−33.3%	−54.1%
45–64	45.5	33.3	28.1	−27.1%	−15.2%	−38.2%	23.7	18.7	18.0	−20.9%	−3.7%	−23.9%
65+	33.5	19.1	17.3	−43.0%	−9.4%	−48.4%	18.4	9.8	9.3	−46.7%	−5.1%	−49.5%
18 and above	43.3	27.1	22.9	−37.4%	−15.5%	−47.1%	27.0	18.4	13.9	−31.9%	−24.5%	−48.5%
**SimSmoke smoking prevalence predictions** b												
18–24	34.3	21.7	19.8	−36.7%	−8.6%	−42.2%	23.9	15.1	13.7	−36.8%	−8.9%	−42.6%
25–44	48.3	27.4	23.9	−43.3%	−12.8%	−50.5%	31.7	17.7	15.2	−44.2%	−14.2%	−52.2%
45–64	45.2	29.2	24.1	−35.4%	−17.6%	−46.7%	23.9	16.9	13.8	−29.3%	−18.2%	−42.0%
65+	34.2	21.5	18.0	−37.1%	−16.6%	−47.5%	18.3	10.6	9.6	−42.1%	−9.7%	−47.8%
18 and above	43.3	26.1	22.6	−39.7%	−13.4%	−47.7%	27.1	16.3	14.0	−39.9%	−14.2%	−48.6%

The prevalence estimates are expressed as rates of current smokers per population, and percentage change values are over time relative to initial values, e.g., “percentage change 1989–2008” represents the difference in prevalence between 2008 and 1989 divided by the 1989 prevalence.

a Survey data are from the 1989 National Survey of Health and Nutrition, the 2003 World Health Survey, and the 2008 GATS.

b Predictions are from the Brazil SimSmoke model as described in the text.

We also compared the predictions of smoking prevalence of those 15 y and above for 2008 to the estimates in the GATS report [32] and found that the predictions for males and females were well within the GATS 95% confidence intervals. The 2008 former smoker rates from SimSmoke also compared well to the rates from the 2008 GATS. For males and females combined, the former smoker rates of SimSmoke versus those of GATS were 10.1% versus 13.8% for ages 25–44 y, 30% versus 29% for ages 45–64 y, and 31% versus 30% for ages 65 y and above (data not shown).

### The Role of Policies in Reducing Smoking Prevalence and Smoking-Attributable Deaths between 1989 and 2010

Tables 3 and 4 show the simulated smoking prevalence and SADs with and without the policies implemented between 1989 and 2010. The counterfactual, with policies at the 1989 levels, has no upper and lower bound since there are no policy effects.

**Table 3:** Smoking prevalence, counterfactuals of no policies changed since 1989 versus policies individually implemented and combined, Brazil SimSmoke, 1989–2050.

Policy Implementation	Year							
	1989	2000	2010	2010 Lower Bound^a^	2010 Upper Bound^a^	2050	2050 Lower Bound^a^	2050 Upper Bound^a^
**Smoking prevalence**								
Counterfactual: all policies at 1989 level	35.4%	32.6%	31.0%			24.9%		
All policies implemented	35.4%	23.7%	16.8%	22.2%	10.5%	10.3%	15.7%	5.6%
**Percent reduction in smoking prevalence from policy change** a								
All policies		−27.4%	−45.9%	−27.8%	−66.4%	−59.1%	−35.9%	−77.9%
Price only		−18.4%	−27.1%	−21.2%	−32.5%	−35.7%	−28.1%	−42.5%
Smoke-free air only		−4.7%	−7.6%	−3.9%	−11.3%	−9.6%	−4.9%	−14.2%
Mass media campaign only		0.0%	−3.5%	−1.8%	−5.3%	−4.5%	−2.3%	−6.8%
Marketing restrictions only		−5.3%	−7.7%	−3.9%	−11.4%	−9.8%	−4.9%	−14.5%
Health warnings only		−0.6%	−4.4%	−2.2%	−6.5%	−6.5%	−3.3%	−9.6%
Cessation treatment only		−1.8%	−5.5%	−1.3%	−24.8%	−9.5%	−4.7%	−19.5%
Youth access restrictions only		0.0%	−0.2%	0.0%	−0.1%	−0.5%	0.0%	−0.8%

a Represents the percent change in prevalence due to a particular policy or all policies relative to the counterfactual with all policies maintained at their 1989 level.

**Table 4:** Smoking-attributable deaths, counterfactuals of no policies changed since 1989 versus policies individually implemented and combined, Brazil SimSmoke, 1989–2050.

Policy Implementation	Year							
	1989	2010	Cumulative 2010	2010 Lower Bound^a^	2010 Upper Bound^a^	Cumulative 2050	2050 Lower Bound^a^	2050 Upper Bound^a^
**SADs**								
Counterfactual: all policies at 1989 level	181,957	283,048	4,998,024			20,401,516		
All policies implemented	181,957	225,048	4,578,810	4,739,196	4,282,963	13,471,388	14,133,046	11,946,994
**Deaths averted from policy change** a								
All policies	—	58,000	419,214	258,828	715,061	6,930,128	4,466,433	10,256,558
Price only	—	30,005	225,275	171,945	276,787	3,770,045	2,937,015	4,541,215
Smoke-free air only	—	9,494	70,742	35,537	105,619	1,173,592	595,340	1,735,314
Mass media campaign only	—	3,657	16,921	8,503	25,255	522,193	263,052	777,483
Marketing restrictions only	—	9,295	69,845	40,149	118,494	1,148,982	591,073	1,726,722
Health warnings only	—	5,041	24,360	12,253	71,380	728,051	368,942	1,795,549
Cessation treatment only	—	8,258	51,349	27,154	98,496	795,916	381,966	1,409,852
Youth access restrictions only	—	—	—	—	—	9,831	—	14,745

a SADs under counterfactual of policies maintained at 1989 level minus SADs with policy (or policies) in place.

Without any of the policies implemented since 1989, SimSmoke projected a slow downward trend in smoking rates, with rates falling from 35.4% in 1989 to 31.0% in 2010 through cessation rates. With all policies implemented, smoking prevalence declined to approximately 17% (lower and upper bounds: 11%–22%), which represents a 46% (28%–66%) relative decline from the 2010 counterfactual level without the implemented policies, and a 52% relative decline from the 1989 level. Similar results were observed for males and females (data not shown). The policy differential continues to grow after 2010, reaching an approximate 59% (36%–78%) relative decline from the simulated 2050 level with policies maintained at their 1989 levels.

As a result of the policies implemented between 1989 and 2010, SimSmoke estimates that a total of 58,000 (35,000–97,000) deaths were averted in 2010 alone. Summing over the years from 1989 to 2010, a total of about 420,000 (260,000–715,000) deaths are averted. The cumulative number of deaths averted increases to almost 7 million (4.5 million–10.3 million) by 2050, of which about 4.5 million are male and 2.5 million are female (data not shown).

**Figure 2:**
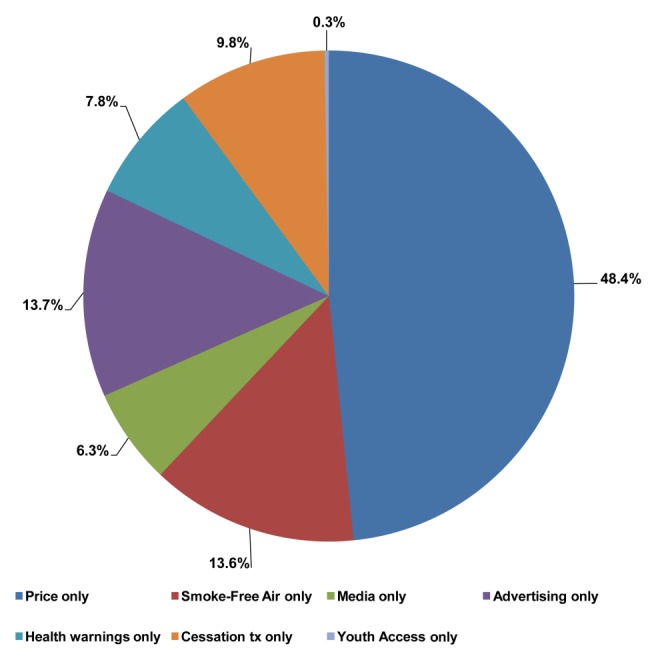
Percent of the reduction in 2010 smoking prevalence* due to individual policies implemented since 1989, estimated by Brazil SimSmoke. Cessation tx, cessation treatment. *Smoking prevalence is for both genders combined.

By examining the effect of each policy implemented between 1989 and 2010 relative to the summed effects, we decomposed the prevalence reductions into component contributions of each policy, as shown in Figure 2. Of the 100% overall reduction in smoking prevalence due to policies implemented by 2010, the percent contributed by tax/price increases was 48%, by stricter smoke-free air laws was 14%, by mass media campaigns was 6%, by stricter marketing restrictions was 14%, by stronger health warnings was 8%, by cessation treatment programs was 10%, and by youth access restrictions was less than 1%. Results were similar by gender except that the relative effects were slightly greater among males for tax increases and among females for cessation policies. In simulation, by 2050, cessation policies and health warnings play a larger role because of their relatively larger effect on cessation rates compared to other policies.

### Role of Policies Implemented in 2011 in Reducing Future Smoking Prevalence and Deaths

We simulated the effect of implementing a stricter set of FCTC-consistent policies maintained from 2011 onward versus a status quo scenario, where policies are maintained at 2010 levels, as shown in Table 5. SADs are summed over the years 2011 to 2050 to serve as an estimate of the SADs of the people alive today.

**Table 5:** Smoking prevalence for ages 18 and above, smoking-attributable deaths, and deaths averted under status quo and FCTC-consistent policy scenarios, Brazil SimSmoke, 2010–2050.

Policy Implementation	Smoking Prevalence					SADs/Deaths Averted^a^		
	2010	2015	2050	Lower Bound 2050	Upper Bound 2050	Cumulative 2011–2050	Lower Bound 2011–2050	Upper Bound 2011–2050
**Smoking Prevalence**								
Status quo	16.8%	15.5%	10.3%			8,892,578	9,513,874	8,749,842
All FCTC policies implemented	16.8%	11.9%	6.3%	7.3%	4.7%	7,563,664	8,657,395	6,783,055
**Reduction in Smoking Prevalence**								
**Independent policy effects**								
Tax at 75% of retail price		−10.2%	−16.7%	−13.0%	−21.5%	469,463	365,730	565,492
Well-enforced smoke-free air laws		−4.5%	−6.4%	−3.1%	−9.5%	268,042	135,972	396,336
Well-enforced marketing ban		−3.0%	−4.8%	−2.4%	−7.2%	171,180	86,231	254,867
High-intensity media campaign		−4.8%	−7.4%	−3.6%	−10.9%	305,436	157,126	459,018
Cessation treatment programs		−2.3%	−4.6%	−2.3%	−9.3%	198,382	100,530	489,257
Well-enforced youth access restrictions		−0.8%	−5.1%	0.0%	−10.1%	28,491	0	42,734
**With all policies implemented**		−23.5%	−38.5%	−29.0%	−54.0%	1,328,914	856,479	1,966,787

a Deaths averted is SADs under status quo policy minus SADs with FCTC-consistent policy (or policies) in place.

If tobacco control policies remain unchanged from their 2010 levels, as in the status quo scenario, the smoking prevalence is projected to decrease to approximately 10% by 2050. From 2011 to 2050, approximately 8.9 (8.7–9.5) million premature deaths in Brazil are attributed to smoking.

Among the available policy measures, tax policy appears especially effective in reducing youth smoking prevalence [33],[34]. With taxes increased from their current level of 60% to 75% of price, smoking prevalence is projected to decline by about 17% (13%–21%) relative to the status quo in 2050, and about 470,000 (366,000–565,000) deaths are averted as a result. By 2050, nationwide smoke-free air laws yield an expected 6% (3%–9%) reduction in prevalence, averting about 268,000 deaths (135,000–396,000); a comprehensive marketing ban yields an expected 5% (2%–7%) reduction in prevalence, averting about 171,100 (86,000–255,000) deaths; a sustained high-intensity campaign yields an expected 7% (4%–11%) reduction, averting 305,400 deaths (157,000–459,000); comprehensive smoking cessation treatment programs yield an expected 5% (2%–9%) reduction, averting about 198,400 (101,000–489,000) deaths; and well-enforced youth access laws yield an expected 5% (0%–8%) reduction, averting about 28,500 (0–43,000) deaths. For youth, deaths are not averted until about 2025, when they reach age 30 y and above.

The final scenario is for all policies combined. By 2050, smoking prevalence would be expected to drop in relative terms by about 39% (29%–54%)—38% for males and 40% for females—relative to the status quo, with upper and lower bounds of approximately 29% to 54%. By 2050, a total of approximately 1,300,000 (860,000–1,970,000) are projected to be averted, of which about 900,000 are male and 400,000 are female.

## Discussion

Smoking prevalence in Brazil has fallen by almost 50% since 1989. Brazil SimSmoke shows that policies played a major role in those steep declines, estimating a 46% relative reduction in prevalence by 2010 above and beyond the reductions that would have occurred in the absence of the policies implemented since 1989. Almost half of the 46% projected reduction is from price increases, with an additional 14% from smoke-free air laws, 14% from marketing restrictions, 8% from health warnings, 6% from anti-smoking mass media campaigns, and 10% from cessation treatment programs. As a result of the policies implemented between 1989 and 2010, SimSmoke estimates that a total of around 420,000 deaths were averted by 2010, increasing to 7 million projected by 2050.

We recommend interpreting the Brazil SimSmoke projections in a conservative manner. The results depend on the reliability of the data and the estimated parameters and assumptions used in the models.

**Figure 3:**
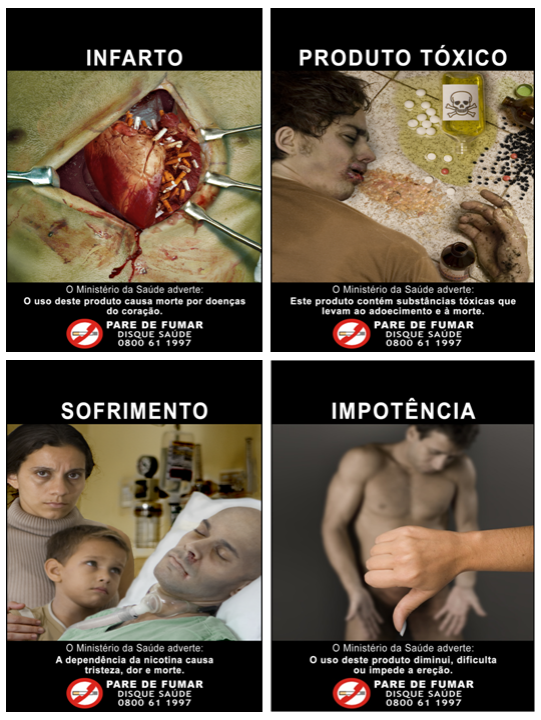
Examples of warnings on cigarette packages in Brazil.

The validation process indicates that SimSmoke predicts less well for two age groups, which may reflect limitations in the model or in the data used to validate SimSmoke. SimSmoke underpredicted the smoking rate reduction for 1989–2008 for those aged 18–24 y. Consistent with the literature, SimSmoke assigns a small value to the impact of warning labels on the young, who are often thought to ignore or resent warnings. However, Brazil has extensively tested their warnings (as portrayed in Figure 3) with younger smokers [35], so that they may have a greater impact than found in previous studies. In particular, health warnings tested well for non-smokers below 24 y and, although aversive with loss-framed content [36], may have reduced initiation. The strong health warnings may have acted synergistically with price increases and stricter smoke-free air laws to obtain the dramatic reductions in smoking initiation since 2003. Additionally, SimSmoke overpredicts the smoking reduction among those aged 45–64 y. Policies, especially those encouraging cessation, may need to be directed at this age group.

SimSmoke does not consider the effect of income on smoking rates. Past studies yield conflicting results on the role of income [37]. Increased purchasing power could influence price elasticities by making cigarettes more affordable [1]. While there are still considerable disparities in income and in access to health care [38], Brazil has made significant efforts to increase the purchasing power of the poor in recent years. Consequently, SimSmoke may overestimate the role of tobacco taxation among lower income segments of the population and underestimate the role of other policy drivers in reducing prevalence.

The estimated relative risks for total mortality of smokers are based on studies from the US [20],[22]–[24],[29],[39], but the risks may differ in Brazil. In particular, differences between Brazil and the US in smoking intensity and duration may influence relative risk. While studies from other middle income nations, including India [39] and Russia [40], obtain a relative risk comparable to that of the US, others such as for Taiwan and Korea [41]–[43] obtain a lower relative risk of about 1.6–1.7. When we used a relative mortality risk of 1.6 rather than the US rate (about 2.1), the number of SADs was reduced by about 35%, and the number of deaths averted due to policies implemented since 1989 was about 4.5 million by 2050 instead of the 7 million projected with US rates. However, the projections do not include the additional deaths averted due to reductions in second-hand smoke exposure and maternal smoking during pregnancy.

Uncertainty regarding individual policy effect sizes was considered for future projections. Uncertainty also arises when more than one policy is implemented. We assumed that the effect of adding a second policy is proportionally reduced if another policy is implemented, but allowed for synergies between media campaigns and other policies. Upon testing for sensitivity, we found that less than 4% of the estimated reduction due to past policies was from synergies with media campaigns. Furthermore, while past studies provide limited guidance on whether implementing multiple policies yields offsetting or synergistic effects [44], some evidence [45] indicates that public policies are synergistic through their cumulative impact on social norms and their reinforcing effects on motivations to quit. These synergies may be especially important in changing the attitudes held by youth and young adults. Nevertheless, with policies modeled as having a unidirectional effect on smoking rates, SimSmoke does not explicitly model feedbacks through social norms and attitudes, and peer and family behaviors.

Many physicians still do not regularly ask their patients whether they smoke, and are even less likely to follow up with advice to quit and suggestions on how best to quit. A US study [30] found that full physician involvement can reduce smoking prevalence by 0.5% in the first year and increase cessation by 10% in future years. These estimates may not be transferable to Brazil, especially because the rural population in Brazil has more limited access to health care. Yet, in one study, although Brazilians with low educational attainment went to health professionals the least (37%), they showed the same level of willingness to quit smoking and received the same level of smoking cessation counseling as individuals with a high degree of education [1].

In Brazil, tobacco control started with large price increases, followed by strong advertising restrictions and health warnings, and, later, partial smoke-free air laws and increased availability of cessation programs. SimSmoke shows that past policies have been very effective in reducing smoking rates, but there is also a strong potential for future policies consistent with the requirements of the FCTC. While Brazil has implemented many strong policies, tobacco control policies could be strengthened to be fully consistent with the FCTC, a legally binding treaty, with the projected effect of decreasing smoking prevalence by as much as 39% by 2050. By implementing these policies, about 1.3 million (out of almost 9 million) deaths could be averted. Low and middle income nations will face major challenges in the years ahead. Brazil's accomplishments demonstrate that, even for a middle income nation, reducing tobacco use is a “winnable battle” that carries huge dividends in terms of reducing mortality and morbidity. Furthermore, most of the measures that Brazil has undertaken cost the government limited resources and, in the case of taxes, generate revenue. Thus, they can help fund programs for those health challenges that have more direct costs, such as infectious diseases, maternal and child health issues, and the provision of basic health services.

## Supporting Information

Text S1 **Mathematical appendix.**
(DOCX)

## Abbreviations

FCTC: Framework Convention on Tobacco Control
GATS: Global Adult Tobacco Survey
SAD: smoking-attributable death
