# Supplementary material for: The Brazil SimSmoke Policy Simulation Model: The Effect of Strong Tobacco Control Policies on Smoking Prevalence and Smoking-Attributable Deaths in a Middle Income Nation
Source: PLoS Med. 2012 Nov 6;9(11):e1001336. doi: 10.1371/journal.pmed.1001336 (PMC3491001; doi:10.1371/journal.pmed.1001336)
Supplement: Text S1 — Mathematical appendix. (DOCX) [file pmed.1001336.s001.docx]

***Brazil SimSmoke*:** **Mathematical Appendix**

The *SimSmoke* model contains a population model, a smoking model, a smoking attributable death model and a series of policy modules for each policy. The model begins with the population in a baseline year divided into current, former, and never smokers. Assuming a discrete, first-order Markov process, population evolves each year through births and deaths, and the smoking population evolves through initiation, cessation, and relapse.

**Demographics Model**

*SimSmoke* is built first on a demographic model. The total population (*Pop)* is distinguished by time period **t** and age **a** (and is further distinguished in the model by gender)**.** Mortality rates (*MR)* are distinguished by age and gender and are based on 1998 mortality rates Newborns depend on first year deaths rates and fertility rates (*Fert)* of females by age with equal birth rates for males and females. Births through the first year (age 0) for each gender are:

*Pop*_t,0_ = 0.5*(1-*MortRate*_0_)* Σ^a^ (*Pop*_t,a,1_ * *Fert*_a,t_), where t=1,…,n; a=14,…,49.

After the first year, the population evolves as:

*Pop*_t,a_ = *Pop*_t-1,a-1_ * (1 – *MortRate*_a_).

The population model does not incorporate immigrations, due to its limited effects in Brazil.

**Smoking Model**

*SimSmoke* divides the population in the base year into (1) never smokers, (2) smokers, and (3) 16 categories of ex-smokers (n=1,…,16+) corresponding to years since last time smoking. After the base year, individuals are classified as never smokers from birth until they initiate smoking or die, as shown by:

*Neversmokers_t,a_ = Neversmokers*_t-1,a-1_ * (1 – *MortRate_a,ns_*)**(1-Initiation rate_a_).*

From never smokers, individuals can become smokers through initiation. Once smokers, they may leave smoking through cessation and return to smoking through relapse. The number of current smokers is tracked as:

*Smokers_t,a_ = Neversmokers*_t-1,a-1_*(1 – *MortRate_a,ns_*)**Initiation rate_a_*

*+ Smokers_t-1,a-1_*(1 – MortRate_t.a,s_)*(1-Cessation rate_a_)*

*+ Σ^16^_n=1_ Ex-smokers_t-1,a-1,n_*(1 – MortRate_t,a,n_)*(Relapse rate_a,n_).*

First year former smokers are determined by the first year cessation rate applied to surviving smokers in the previous year. After the first year quit, individuals who have been former smokers for between n= 2,…, 15 are defined as:

*Former smokers_t,a,n =_ Former smokers_t-1,a-1,n-1_*(1 – MortRate_a,n_)*(1-Relapse rate_a,n-1_).*

For those who have ceased smoking for more than fifteen years, we add to the above equation the number of former smokers from the previous year who have quit for more than fifteen years and have not died or relapsed in the previous year.

**Smoking Model Assumptions***:*

Initiation

- Initiation depends on current and not past initiation rates.
- Initiation rates vary by age and gender

- Initiation rates are measured by changes in baseline prevalence from age to age through ages 29, they incorporate initiation minus cessation, thus reflecting net initiation.
- Initiation rates vary by age and gender
- Initiation rates are assumed constant over time in the absence of changes in policy.

Cessation

- Quitting behavior only depends on current cessation rates and do not depend on past cessation behaviors
- Cessation rates vary by age and gender
- Cessation rates allow for the transition from current to former smokers and are assumed constant over time subject to changes in policy.
- Cessation rates are applied from age 29, based on the assumption that cessation before that age has limited effects on future health.
- Cessation rates are assumed constant over time in the absence of changes in policy.

Relapse

- Former smokers (FS) and relapse rates vary by age and gender and years quit.
- Relapse rates are assumed constant over time, independent of changes in policy. They do not depend on past cessation behaviors.

**Smoking-Attributable Death Model**

Using standard attribution formulas (Shultz et al. 2000), smoking-attributable deaths are estimated for each age and smoking group by multiplying the number of smokers in that group by the difference between the death rate of that smoking group and the death rate of never smokers. To estimate the age and smoking group specific death rate *DR_a,s_*, we used the age and gender specific prevalence (*Prev*), relative risks (*RR_a,s_*) and death rates (*DR*_a_). The death rate of an age group can be expressed as:

*DR*_a_ = *PrevNeversmokers*_a,ns_**DR*_a,ns +_ *PrevSmokers*_a,s_**DR* _a,s_ + Σ^n^ (*PrevEx-smokers*_a,n_ **DR* _a,n_),

Dividing both sides by *DR*_a,ns_, we obtain:

*DR*_a_ /*DR*_a,ns_ = *PrevNeversmokers*_a,ns +_ *PrevSmokers*_a,s_**RR* _a,s_ + Σ^n^ (*PrevEx-smokers*_a,n_ **RR* _a,n_),

because *RR_a,s_= DR_a,s_/DR_a,ns_* and similarly for ex-smokers, and *RR _a,ns_*=1. Rearranging terms, the death rate for never smokers becomes:

*DR_a,ns_ = DR*_a_ /[*PrevNeversmokers*_a,ns_ + *PrevSmokers*_a,s_**RR* _a,s_ + Σ^n^ (*PrevEx-smokers*_a,n_**RR* _a,n_].

For any smoking group s^*^ (of either smokers or ex-smokers), we multiply both sides by *DR_a,s*_ / DR_a,ns_* to obtain the death rate:

*DR_a,s*_ = DR_a_* RR _a.s_** /

[PrevNeversmokers_a,ns_+PrevSmokers_a,s_*RR _a,s_+Σ^n^ (PrevEx-smokers_a,n_*RR _a,n_)].

**Smoking-Attributable Death Model Assumptions:**

- Current, former or never smoker deaths are derived using a formula that depends on the relative risks of total mortality of current smoking and of former smoking (distinguished by years quit) and smoking prevalence rates in 1989. The relative risks are based on the CPS-II,
- The relative risks are assumed to be constant over time.

**Policy Effects**

The effects of policies are calculated as percent reductions, PR, relative to the initial rates, i.e., [PR= (post-policy rate - initial rate)/initial rate, where PR< 0]. Policies generally have the greatest effect in the first years. The effects are modeled as a constant proportional effect on smoking prevalence in the first year that the policy is implemented, i.e., *Smokers_t,a_ * (1+PR_i,t,a_)* for policy **i** at time period **t** and which may vary by age **a**.

After the first year, policies affect initiation and cessation rates. If the policy affects initiation, the effects of the policy are sustained through lower initiation rates. Throughout the years in which the policy **i** is in effect, the percentage reduction lowers the initiation rate by *(1+PR_i,a_).* The effects of a policy **i** may also be augmented over the same time period through increases in the first year cessation rate by *(1-PR_i,a_).* First-year quit rates remain elevated for each of the policies (except youth access policies), as justified by the higher propensity to quit among individuals who smoke less as a result of policies and other factors (e.g., economic and informational) creating incentives to quit. It is assumed that the proportion of individuals who relapse increases in direct proportion with any added cessation, implying that the rates of relapse are unaffected by policy changes. Thus, policies have their greatest affect on cessation (directly through the prevalence rate) in the first year that the policy is in effect. Each of the policies also continues to affect initiation and first-year cessation rates during the period over which a policy is in effect.

When more than one policy is in effect, there may be synergies built into the model as described below. Otherwise, it is generally assumed that there are constant proportional reductions, i.e., *(1+PR_i_)*(1+PR_j_)* for policies **i** and **j**. This formulation implies that the relative effect of a policy is independent of other policies in effect, but the absolute reduction is smaller when another policy is in effect (due to the reduction in the smoking rate from the other policy).

Direct modifications are made in the policy effects for individual countries, especially as they relate to LMICs (such as Brazil) relative to HICs. Two main types of adjustments are made using scale factors that pertain primarily to LMICs. The first, **URBAN** (> 0, < 1), is for the degree of urbanization and is generally meant to capture difficulties in reaching populations that are in more rural areas. This variable is measured as 1-the percent of economy in rural trades [agriculture] in a country/percent of economy in rural trades). The other scale factor, **AWARENESS** (> 1) is for the potentially greater impact of awareness about the dangers of smoking in countries where such information is at a lower base level than in the U.S. The level of **AWARENESS** depends on the policy, as designated below. The level of **AWARENESS** is applied to LMICs where information is less well-disseminated than in HICs. These scale factors are applied multiplicatively, so that the effect sizes become *(1+URBAN_i*_ AWARENESS_i *_PR_i,a_)* for the prevalence and initiation effects and *(1+URBAN_i*_ AWARENESS_i *_PR_i,a_)* for the cessation effect.

**Policy Models** **Assumptions:**

- The policy effects are modelled as constant percentage reductions, through prevalence in the first year of implementation, and generally through initiation and cessation in future years.
- When more than one policy is applied, the effects are assumed to be multiplicative through their respective percentage reductions. An exception is synergies (as described above) through publicity from media campaigns.
- Policies as they have implemented are applied to the tracking period (1989 to 2010).

In the absence of future policy change, future smoking prevalence and smoking-attributable deaths are projected assuming that policies are assumed to remain constant after 2010 at their levels in 2010.

**Taxation Policies**

The tax policy module in *SimSmoke* follows the MPOWER Report and specifies the tax in percentage terms relative to the retail price. In keeping with the recent recommendations under MPOWER, we consider the effect of increasing excise taxes to 70% of the retail price.

The taxation effect works through price. In *SimSmoke*, the effect of price depends on price elasticities (obtained from relevant studies), which are assumed to be constant in *SimSmoke*. The formula for constant price elasticity, ***E***, is defined in terms of the price, **P**, and quantity, **Q**, both distinguished by their new level, designated by subscript **n**, and their initial level, designated by subscript **t**. The arc elasticity formula is written as:

***= [(Q_t+1_ – Q_t_ )/(Q_t+1_ + Q_t_)]/[ (P_t+1_– P_t_)/(P_t+1_ + P_t_)], < 0.***

To solve for ***Q_t_***, the equation is rewritten as:

***[(Q_t+1_ – Q_t_ )/(Q_t+1_ + Q_t_)] =E*[ (P_t+1_– P_t_)/(P_t+1_ + P_t_)]***

Denoting ***E* [ (P_t+1_– P_t_)/(P_t+1_ + P_t_)]*** *by Δ,* the equation can be solved for

***Q_t+1_ – Q_t_ = Δ( Q_t+1_ + Q_t_),* or**

***Q_t+1_ = Q_t_ *(1 + Δ)/(1 - Δ)****.*

Since we focus on participation rates, **Q** translates to the number of smokers for relatively small changes in population. In order to distinguish the effects for different periods, let **Δ_t_** denote the effects of a price change between periods **t** and periods **t-1**. Let ***δ_t_ = (1+Δ_t_)/(1-Δ_t_)****,* then

***Q_t+1_ = δ_t_ * Q_t._***

The elasticities vary by age based on U.S. data (as described in the text) and are scaled by country relative to the U.S., usually in terms of overall elasticity (participation and conditional quantity), depending on the type of elasticity available. For example, the average overall elasticity for the U.S*.* is 0.4 [[1](#_ENREF_1)].

Actual prices adjusted for inflation are used for the tracking period. Future price changes occur through tax increases, which are specified as a portion of price. Future inflation adjusted prices are assumed constant in the absence of a tax change.

The model assumes that prices increases in absolute terms with the amount of the cigarette tax, based on Sung [[2](#_ENREF_2)] and evidence from other countries [[3](#_ENREF_3)]. Let ***T_t_*** be the tax rate as expressed as a percent of price in period **t**. To derive future prices, we first estimate the price net of taxes, **PNT,** as the retail price, **P_t_,** multiplied by (**1-*T***) for the last year before projection. Assuming that the factory price (after adjusting for inflation), the import and value added tax and the average percent markup by the manufacturers and foreign tobacco firms remains constant, then the amount of the total tax will be equal to ***T*/ (1-*T*)** times the price net of taxes [[4](#_ENREF_4)]. Then the retail price can be re-written:

**P_t_ = PNT *+* PNT** ******T_t_ /(1-T_t_****)*,

where the second term is the amount of the price that is taxed. We assume that the net of tax price remains constant and that prices increase by the amount of the tax, so that the new price is obtained by substituting the new tax ***T_t +1_*** for ***T_t_*** in the second term.

Taxes on cigarettes may include value added, import and excise taxes. We do not consider import taxes in our analysis, since they are a small percent of price. Excise taxes apply only to cigarettes and may be implemented in percentage terms, i.e., ad valorem taxes, or fixed amounts per unit, i.e. specific taxes. In our analysis, the value added tax (in percentage terms) is held constant, since it applies to all goods, not just cigarettes, and it is applied to excise taxes as well as the price net of taxes. Consequently, it amplifies the effect of an excise tax increase, so that the excise tax as a percent of price diminishes when price adjusts to a new level. Consequently, we consider two cases: 1) where the excise tax is increased to 70%, but after adjustment of the value added tax no longer equals 70%, and 2) where the excise tax is further adjusted, after allowing for the effect of the value added tax, so that it equals 70% of the final retail price. When the effect of the value added tax is incorporated, the value added tax is included in **T_t_** in calculating the price net of tax and amount of the tax. To exclude the amplifying effect of total taxes, we only consider excise taxes in **T_t_** in calculating both the price net of tax and amount of the tax.

**Smoke-Free Air Policies**

Three types of smoke-free air policies (worksite, restaurant, and bars) are included in MPOWER *SimSmoke*, with the effect of worksite further distinguished by its stringency. Worksite bans are distinguished corresponding to data provided in the MPOWER Report as: 1) partial as designated by a ban in 2 of the 3 types of facilities: health, university, and government facilities, 2) ban in indoor offices only, and 3) ban in all indoor workplaces (including offices and other indoor workplaces, universities, and government). These policies are cumulative, i.e., inclusive of the previous policy, with the MPOWER target policy a complete ban. Consistent with the MPOWER Report, the model distinguishes only a total restaurant ban and a total ban in bars and restaurants. The MPOWER model includes a ban in pubs and bars. The model includes bans in other public places, based on the corresponding MPOWER variables, public transport, and supplementary information. An enforcement index is based on the MPOWER Report, whereby enforcement is scored between 1 and 10, with 10 as the highest level. In addition, publicity is directly dependent on the level of tobacco control campaigns.

For LMICs, the effects are adjusted downward by using the **URBAN** index to account for the percent of the population not affected due to lower rates of labor working in indoor workplaces and increased by 50% in a country in an LMIC without previously active tobacco control (then **AWARENESS** = 1.5, and otherwise = 1). In addition, while half of the effects occur automatically through passage of the law (e.g., due to a change in norms), the other half of the effects depend on enforcement (0<**ENF**<1, using the MPOWER index=1,…10 divided by 10 to be scaled to 1) and a publicity index based on tobacco control spending (= .5 if no tobacco control spending is low or non-existent, 0.75 if tobacco control spending is medium, and 1 if tobacco control spending is high). Letting *SFL’* equal the effect size of type **k** for a HIC with high enforcement and publicity, the effect *SFL_i_* for country **i** is:

***SFL_i,k_ = SFL_~~k~~_’ * URBAN_i_ * AWARENESS_i_ *0.5 (1+ENF_i_*PUB_i_).***

**Marketing Restrictions**

Four levels of marketing restriction policies are distinguished: none, minimal, moderate, and comprehensive. The effects differ for prevalence, cessation, and initiation, and also depend on enforcement.

While a lower degree of urbanization may reduce the effectiveness of advertising by making it more difficult to reach those in rural areas, Blecher [[5](#_ENREF_5)] found that the effects of comprehensive bans are at least twice as great in LMICs as in HICs. In the model, this phenomenon is attributed to lack of awareness of the dangers of smoking. The effect in the LMICs is doubled relative to HMICs. As for smoke-free laws, a total lack of enforcement reduces the impact by half (0<**ENF**<1, using the MPOWER index = 1…10 divided by 10 to be scaled to 1). For marketing restrictions at a level **k** for HICs with high enforcement designated as MR’, the effect for country **i** will be:

***MR_i,k_ = MRK_k_’ * AWARENESS_i_ *0.5 (1+ENF_i_)****.*

**Health Warnings**

The MPOWER *SimSmoke* distinguishes four levels of policy (none, mild, moderate, and strong) and the effects depend on the awareness factor. Because this policy is geared toward the dangers of smoking, the level of initial awareness is expected to play an important role. For LMICs, the effects are doubled due to the lower initial level of awareness (**AWARENESS** =2 in that case, **AWARENESS**= 1 otherwise). For effect size *HW*’ at level **k** for health warning in a HIC, the effect for country **i** is:

***HW_i,k_ = HW_k_’ * AWARENESS_i_.***

**Tobacco Control Campaigns**

Tobacco control campaigns are specified: high, medium, and low. The degree of urbanization affects the ability to reach rural populations through the media and even local campaigns, and is taken into consideration in examining the effect of tobacco control spending. However, the level of awareness is expected to be low in LMICs that have not had prior policies, and for those countries is accorded a value of 1.5. For tobacco control spending at level **k** at effect size *TC’* in a HIC, the effect for country **i** is:

***TC_i,k_ = TC_~~k~~_’ * URBAN_i_ * AWARENESS_i_***

**Cessation Treatment Policies**

The new PT availability sub-policy option corresponds to the information in the MPOWER Report regarding whether nicotine replacement treatment (NRT) and/or Buproprion are available and where they may be obtained. The availability indicators are first developed separately for each PT by setting them equal to:

PT1 = 2 if NRT is yes, 0 if no

PT2 = 1 if Buproprion is yes, 0 if no

When pharmacotherapy is available, the MPOWER Report distinguishes whether each PT is available in a general store or pharmacy and if a prescription (Rx) is required. We do not distinguish effect sizes (they are each assigned a value 1) by these sources except when NRT is only available by prescription. Since access is thus more limited, the NRT variable is multiplied by 0.5, indicating that the effect is reduced by 50%. To get an overall effect, the indicators for NRT and Buproprion (PT1 and PT2) are summed and divided by 3 to obtain an overall indicator with a value between 0 and 1 that is used to scale the percentage effect of the new treatment availability sub-policy. If the value of the sub-policy is 1 (the policy is effect in full), then prevalence is reduced by 1.0% in the first year of the policy (which is roughly equivalent to the effect of 15% of smokers using either or both of the PTs with a 10% average success rate net of relapse in the first year) and the pre-policy cessation rate is increased by 6% in all years after the first (equivalent to quit attempts increased by 30% due to new PT users with those users having a 20% first year success rate). Therefore, there is no effect on initiation.

Treatment coverage focuses on where the treatment is provided. We followed the MPOWER Report that distinguishes place of provision of cessation treatments by the following: primary care facilities, hospitals, offices of health professionals, community and other. For each location, we designate a value of score for each of the above locations: 0 = None, 1 = Yes in some, and 2 = Yes in most. We then sum the scores. The highest possible score is 10, but a full effect is designated if at least 4 of the 5 places have indicated yes, whereby a score of 8 is for the full effect. To scale to 1, we multiply by 0.125 (1/8). That indicator is used to scale the effect of the financial coverage of treatment sub-policy.

The effect of treatment coverage depends on publicity. The effect of publicity on financial coverage of treatment is (1- 0.25 *(1- publicity)); where publicity = 1 if a high level tobacco control campaign, 0.5 if medium level, and 0.25 if low level)) so that the effects of treatment availability are scaled as much as a 25% reduction by this variable if publicity is less than high (equivalent to an additional 15% of smokers using treatment with a 15% success rate net of relapse) in the first year of the policy and the cessation rate is increased by 12% (equivalent to quit attempts increased by 40% with the new treatment users having a 30% first year success rate) in all years after the first.

In the MPOWER Report, quitlines are distinguished only by whether the population has access to a toll free quitline. The effect of quitlines also depends on publicity, which uses the same equation as for the financial access sub-policy. If an active quitline with follow-up is implemented and the program is well publicized through a tobacco control campaign, then prevalence is reduced by 0.75% (equivalent to 5% of smokers using the quitline with a 15% success rate net of relapse) in the first year of the policy and the cessation rate is increased by 7.5% (quit attempts increased by 25% with users having a 30% first year success rate) in all future years.

When more than one of the sub-policies is implemented, the effects are additive with the following exceptions. When all sub-policies are implemented, smoking prevalence is reduced by 4.75% and the first year cessation rate is increased by 39.3%. We allow for less effect if the country is rural because of less access to health care in rural areas, but we allow for a 50% greater effect in countries where awareness of health dangers is low. Thus, with effect size *CTP*’ for cessation treatment policies in an HIC, the effect in country **i** at level kis

***CTP_i,k_ = CTP_~~k~~_’ * URBAN_i_ * AWARENESS_i_***

**References**

1. Jha P, Chaloupka F, editors (2000) Tobacco control in developing countries. New York: Oxford University Press.

2. Sung H, Hu T, Keeler T (1994) Cigarette taxation and demand: An empirical model. Contemporary Economic Policy 12: 91-100.

3. Chaloupka FJ, Hu T, Warner KE, Jacobs R, Yurekli A (2000) The taxation of tobacco products. In: Jha P, Chaloupka F, editors. Tobacco control in developing countries: Oxford University Press. pp. 237-272.

4. Sarntisart I (2003) An Economic Analysis of Tobacco Control in Thailand. Washington, D.C.: World Bank. Economics of Tobacco Control Paper No. 15 Economics of Tobacco Control Paper No. 15.

5. Blecher E (2008) The impact of tobacco advertising bans on consumption in developing countries. J Health Econ 27: 930-942.
